# Impressive and Prolonged Response with Lenvatinib in a Highly Pretreated Patient with Metastatic Clear Cell Renal Cancer: A Case Report

**DOI:** 10.15586/jkcvhl.v11i2.317

**Published:** 2024-04-10

**Authors:** Sara Demurtas, Mara Frascaroli, Federico Sottotetti, Alessandro Rametta, Giuseppe Procopio, Laura Deborah Locati

**Affiliations:** 1 Internal Medicine and Therapeutics Department, University of Pavia, Pavia, Italy;; 2 Medical Oncology, Maugeri Clinical Research Institutes IRCCS, Pavia; Italy;; 3 Medical Oncology, Fondazione IRCCS Istituto Nazionale dei Tumori, Milan, Italy

**Keywords:** case report, lenvatinib, renal cell carcinoma, tyrosine kinase inhibitor, VHL mutation

## Abstract

Clear cell renal carcinoma (ccRCC) can occur in young people and could be associated with an aggressive behavior. While for the first-line treatment in metastatic disease, there is an agreement to rely on an immunotherapy (IO)-based combination regimen, no standard second-line regimens exist. Generally, tyrosine kinase inhibitors (TKIs) are employed, even in sequence, although no trials have demonstrated yet the best succession. Herein, we present the case of a 39-year-old male, with a very aggressive ccRCC with somatic *VHL* mutation and distant metastases at diagnosis. He was treated with four different lines of therapies, including TKIs, with progressive multiple tumor deposits. Lenvatinib alone as the fifth line was able to induce a remarkable and prolonged tumor shrinkage with manageable toxicities.

## Introduction

Kidney cancer is the sixteenth most common cancer worldwide with approximately 430,000 new cases and 180,000 deaths in 2020, accounting for 2% of all new cancer diagnoses (1). The statistics include renal cell carcinoma (RCC), which represents the majority of kidney cancers, and urothelial cancer of the renal pelvis. Clear cell variant (ccRCC) accounts for approximately 75% of RCCs; the remaining 25% corresponds to more uncommon subtypes, with different histological and molecular profiles (2). Genomic alterations (loss-of-function deletion/frameshift) of the von Hippel-Lindau gene (VHL) occur in about 70%–80% of sporadic ccRCC (COSMIC v96), representing an early event in cancerogenesis (3,4). Angiogenesis acts as a main biological driver in RCC development, indeed VHL loss causes dysregulation of the VEGFR pathway, accumulation of hypoxia-inducible factor (HIF), and transcription of pro-angiogenic growth factors. These specific molecular findings were the background for the development of anti-angiogenic treatments which, up to now, represent the standard of care for patients with advanced and/or metastatic disease. Therapeutic approaches in these latter cases may vary according to the prognostic risk groups, based on the International Metastatic RCC Database Consortium (IMDC) prognostic model (5,6). Except for patients with synchronous distant metastases who can be evaluated for cytoreductive nephrectomy, patients with distant metastases are candidates for systemic therapy (7). Since the approval of nivolumab and ipilimumab, except for contraindications, the upfront treatment consists ofan immune checkpoint inhibitor (ICI)-based combination regimen, such as ICI/ICI (ipilimumab/nivolumab) or ICI/tyrosine kinase inhibitor (TKI) (7), including axitinib/pembrolizumab, cabozantinib/nivolumab, and lenvatinib/pembrolizumab (7). The ipilimumab/nivolumab regimen is indicated for intermediate and poor-risk patients, while the others can be administered regardless of the IMDC prognostic group (7). The use of monotherapy with antiangiogenic agents is preferred in the subsequent lines or patients ineligible for ICI. Generally, first-generation of TKIs, such as sunitinib or pazopanib, is recommended in patients with IMDC favorable-risk disease, whereas cabozantinib is preferable in intermediate and poor-risk patients (7). Subsequent sequences of treatment depend on the previous choices (7). The probability of response in patients with advanced/metastatic disease decreases with the increase in the number of treatments. Herein, we report the case of a young patient with a synchronous metastatic ccRCC, obtaining a prolonged and major clinical response to lenvatinib alone, after four previous lines of therapies.

## Case Report

In July 2019, a 39-year-old man with a history of paranoid schizophrenia and hypertension was admitted to the emergency department for dyspnea and desaturation (oxygen saturation was 77% while the patient was breathing ambient air). Some days before the admission, the patient had developed a fever and cough, resistant to antibiotic therapy. A whole-body computed tomography (CT) revealed a lytic lesion in the right zygomatic region (≈ 20 mm), bilateral multiple lung nodules (max 17x15 mm in the right lower lobe, RLL), bilateral endobronchial lesions, mediastinal (max 12x12 mm,) and lumbo-aortic (max 16 mm) lymphadenopathies and bilateral renal multiple nodules (max 82x66 mm in the left kidney, 59x51 mm in the right kidney). A bronchoscopy was performed to reduce the obstruction and to take a biopsy. The histopathological examination revealed a poorly differentiated clear cell carcinoma, consistent with renal primitivity. A core biopsy of the zygomatic lesion showed a poorly differentiated clear cell carcinoma, suspicious for deposit from RCC. The patient was classified as poor-risk according to IMDC, but at that time the ICI-based combinations were not approved in Italy yet and he started Cabozantinib 60 mg daily as first-line treatment in August 2020. After 2 months, a whole-body CT scan showed a stable disease (nodule in RLL: 13 mm; left kidney nodule: 70 mm; right kidney nodule: 6.6 cm), he continued on Cabozantinib 60 mg daily, reporting side effects of fatigue and a worsening of hypertension, both classified as G2 according to CTCAE v5.0. In April 2021, the whole body CT showed a partial response according to RECIST v1.1 in both kidneys nodules, stable disease in the lungs and the right zygomatic bone, and a disease progression with the appearance of multiple new liver lesions (max 31 mm in the IV segment), new left adrenal gland (nodule of 15 mm), and new bone lesions (T10, T11, and sternum). Cabozantinib was withdrawn and a second-line treatment with nivolumab flat-dosing (240 mg every 2 weeks) was started in April 2021. The CT scan performed after 3 months showed complete regression of the left adrenal gland nodule, a partial response of the liver nodules (max 17 mm in the IV segment), kidney nodules (longest diameter 5 cm in left kidney and complete disappearance of the right kidney nodes), lungs nodules (<1 cm) and T10, T11, and sternum lesions; whereas, the right zygomatic lesion progressed (longest diameter 25 mm), involving the soft tissues and the omolateral orbit. In August 2021, the patient received palliative radiotherapy with volumetric modulated arc therapy (VMAT) technique on the right orbital region (20 Gy) and continued on nivolumab until November 2021, when a very fast-growing lesion appeared on the tip of the tongue, requiring a palliative partial glossectomy. Pathological report confirmed a poorly differentiated ccRCC, with sarcomatoid features, R0, consistent with the renal primitivity. In December 2021, the tongue lesion recurred and a new whole-body CT showed a progression of the disease in the right zygomatic region (40x32 mm), lung (13 mm), and left kidney (75x55 mm) with stable disease in the other sites. In the same month, the patient started sunitinib 50 mg daily (4 weeks on/2 weeks off); it was very well tolerated, without reporting adverse events with a complete regression of the tongue lesion. After 1 month, due to the rapid enlargement of the periorbital right lesion conditioning exophthalmos, a rechallenge with cabozantinib at the dose of 40 mg daily was attempted. One month later, a further progression was observed with the whole body CT scan at the right zygomatic lesion (50x40 mm), lung nodes (max 16 mm in the right upper lobe, RUL), and a new pleural lesion (new pleural thickening, 8 mm). FoundationOne®CDx test done on the tongue lesion, revealed a somatic VHL gene mutation, variant L89R (c.266T>G). At this point, we decided to switch to a different antiangiogenetic agent. In March 2022, we started lenvatinib 20 mg daily off-label (after obtaining IRB approval), observing a rapid tumor shrinkage in the first 2 weeks of treatment, indeed the patient was able to open his right eye again. After 2 months (May 2022), a CT scan confirmed the partial response of the right zygomatic lesion (37x30 mm), reduction in the lung nodules (max 9 mm in RUL), pleural thickening (5 mm), and stable disease in the left kidney node and the other bone lesions. In July 2022, a G3 diarrhea occurred and lenvatinib was temporarily withdrawn until regression of the toxicity to G1; then, lenvatinib was restarted at the same dosage. The subsequent whole-body CT performed after 3 months (August 2022) showed a further regression of the orbital lesion (25x18 mm), an almost complete response of the lung nodules with the persistence of a 2 mm lesion in the RUL and a stable disease in the other sites. In November 2022, the G3 diarrhea occurred again, with subsequent lenvatinib temporary withdrawal, resulting in the resolution of the adverse event. From there, lenvatinib was restarted at a dose of 14 mg per day; since then, no more clinically significant diarrhea occurred. The following whole-body CT was carried out in November 2022 and demonstrated an additional small reduction of the lesion in the orbital and right zygomatic region (24x16 mm), the complete response of the lung parenchyma, a stable disease of abdominal and bone lesions, but described also the appearance of necrotic lymphadenopathy in left lung hilum (22 mm). The next CT (March 2023) showed a further reduction of the right orbital and zygomatic lesion (20x12 mm), confirmed the complete response of the lung parenchyma and the stability of right kidney and bone (T11) lesions, but described the dimensional progression of the lymph node in left lung hilum (25 mm), of the left kidney lesion (63 mm vs. 48 mm) and the appearance of para-aortic lymphadenopathy. Considering the oligoprogression, the excellent response of right orbital/zygomatic and lung lesions, the clinical benefit and the previous therapeutic lines, we decided to continue lenvatinib, proposing the patient for radiotherapy. In May 2023, he underwent stereotactic radiotherapy with the VMAT technique to the left kidney lesion and para-aortic node (30 Gy) and to T11 lesion (30 Gy). Lenvatinib was suspended 3 days before, during radiotherapy and resumed 3 days after the end of radiotherapy. Then the patient continued lenvatinib 14 mg daily until August 2023, when he developed a very rapid lung progression, conditioning a progressive respiratory failure until death. At that time (August 2023), the complete regression of the right orbital lesion was still ongoing.

The patient gave his consent to report his clinical history in this article.

## Discussion

Clear cell renal carcinoma (ccRCC) can occur in young people (about 9% present before the age of 45) (8) and it could be associated with rare and atypical sites of metastases. The prognosis of renal cancer has radically changed since the ICI-based combinations (ICI/ICI or ICI/TKI) have become the standard of care in the frontline setting, indeed the median overall survival (mOS) was 8.5 months when RCC was treated with interferon-α (9), increasing up to 26.4 months with sunitinib as first-line treatment (10), 45.7 months with pembrolizumab and axitinib (11), 55.7 months in patients treated with ICI/ICI combination (12), 49.5 months with nivolumab and cabozantinib (13), and 53.7 months in patients treated with pembrolizumab and lenvatinib in upfront setting (14). When ccRCC becomes refractory, the treatment choice remains more uncertain. Usually, it proceeds with a TKI monotherapy, for example, cabozantinib, sunitinib, or tivozanib. The METEOR trial has compared cabozantinib versus everolimus in patients previously treated with one or more TKIs (15); the mOS was superior in patients receiving cabozantinib (21.4 vs. 16.5 months; hazard ratio (HR), 0.66; 95% CI 0.53–0.83; p=0.00026), with an objective response rate (ORR) of 17% for cabozantinib versus 3% for everolimus. Adverse events (AEs) >Grade 3 were recorded in 71% of patients in the cabozantinib group and 60% of patients treated with everolimus, being the most frequent hypertension, diarrhea, fatigue, and palmar-plantar erythrodysesthesia syndrome (15). The BREAKPOINT trial has demonstrated the activity of cabozantinib as second-line after ICIs, showing a mOS of 13.8 months (95% CI 7.7–29.0) and an ORR of 37.9% (16). A real-world study conducted by Wells et al. on the effectiveness of second-line sunitinib after ICI therapy showed a mOS of 15.6 months (95% CI 9.8–21.7) and an ORR of 22.5% (17). In the trial that demonstrated the superiority of sunitinib over interferon alfa (IFN-α) (10), the most common Grade 3 adverse events were hypertension, fatigue, diarrhea, and hand-foot syndrome. In the TIVO-3 trial, tivozanib was superior to sorafenib in terms of mPFS (5.6 months vs. 3.9 months; HR 0.73; 95% CI 0.56–0.94; p = 0.016) and ORR (18% vs. 8%) in a patient population heavily pretreated with two or more lines (18). Treatment-related adverse events occurred in 84% of patients treated with tivozanib and in 94% of patients treated with sorafenib; the most common Grade 3 adverse events of tivozanib-related were hypertension, fatigue, decrease of appetite, and diarrhea. No Grade 4 events were reported (18). Lenvatinib has been studied alone (24 mg/day) or in combination with everolimus (lenvatinib 18 mg/day and everolimus 5 mg/day) versus everolimus alone (10 mg/day) as second-line treatment (19). Lenvatinib plus everolimus improved the mPFS (14.6 vs. 5.5 months; HR 0.40; 95% CI 0.24–0.68; p=0.0005), mOS (25.5 vs. 15.4; HR 0.51; 95% CI 0.30–0.88; p=0.024), and ORR (43% vs. 6%) over everolimus alone. The mPFS was also prolonged with single-agent lenvatinib (7.4 months) with respect to everolimus (HR 0.61; 95% CI 0.38–0.98; p=0.048); the mOS and ORR were 19.1 months and 27%, respectively, for lenvatinib alone. The mPFS (HR 0.66; 95% CI 0.39–1.10; p=0.12) and mOS (HR 0.75; 95% CI 0.43–1.30; p=0.32) were not different between patients who received the combination lenvatinib plus everolimus versus the single-agent lenvatinib. Serious AEs > Grade 3 occurred in 38%, 44%, and 45% of patients treated with everolimus, lenvatinib and lenvatinib plus everolimus, respectively. Diarrhea, fatigue, and hypertension were the most common > Grade 3 AEs for lenvatinib plus everolimus; proteinuria, hypertension, and diarrhea for lenvatinib; anemia, dyspnea, hyperglycemia, and hypertriglyceridemia for everolimus, respectively (19). Table 1 summarizes the efficacy data of the TKIs for the treatment of RCC in further lines after at least one previous VEGF-target therapy.

**Table 1:** Efficacy of TKIs in further lines for mRCC.

	Cabozantinib (15)	Sunitinib (17)	Tivozanib (18)	Axitinib (29)	Lenvatinib (19)
ORR (%)	17	22.5	18	23	27
mPFS (mo)	7.4	–	5.6	8.3	7.4
mOS (mo)	21.4	15.6	16.4	20.1	19.1

A single-center retrospective analysis was conducted on 55 RCC patients treated with lenvatinib ± everolimus (42 patients received the combination and 13 lenvatinib alone) after progression on, at least, two lines (one ICI and one TKI) (20). Patients were heavily pre-treated, as the median number of previous therapies, was 4. ORR was 21.8%, with one complete response and the mOS from initiation of lenvatinib ± everolimus was 12.1 months (95% CI 8.8–16.0).

**Figure 1:**
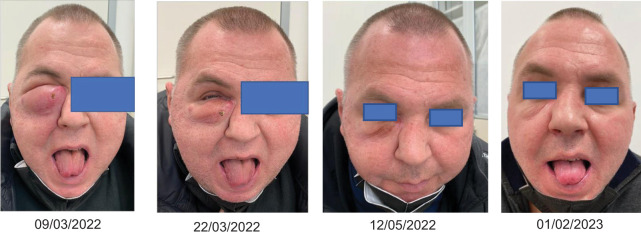
Tumor shrinkage obtained with lenvatinib over the time.

Our patient was young at diagnosis and presented a very aggressive ccRCC with distant metastases; the FoundationOne®CDx test revealed a somatic VHL gene alteration. Somatic VHL mutations (loss-of-function or frameshift) or VHL promoter methylation occur frequently in ccRCC, being reported in about 50% of cases (3). VHL gene encodes for the VHL protein (pVHL), which has multiple functions, including the proteolytic degradation of the HIF family by binding to its α subunits. In normoxic conditions, when HIF is hydroxylated, it is recognized by VHL and so then degraded. When VHL is altered, HIFα accumulates in the cancer cells activating the downstream signaling and its target genes, including the vascular endothelial growth factor (VEGF), and other growth factors or their receptors involved in the angiogenesis process (plateletderived growth factor B, PDGF), cell proliferation and survival (transforming growth factorα, TGFα, and epidermal growth factor receptor, EGFR) (21). To date, clinical trials have failed to show a correlation between VHL gene status and prognosis or treatment outcomes (22–25), whereas angiogenesis maintains a pivotal role in ccRCC cancer progression. Lenvatinib is a second-generation TKI, a potent VEGFR2 and FGFR inhibitor. The inhibition of this latter pathway, in particular, distinguishes lenvatinib from the other TKIs, representing the FGFR as one of the well-known mechanisms of escape to VEGF/VEGFR inhibitors (26). Compared to sunitinib and sorafenib, lenvatinib carries the highest half maximal inhibitory concentration (IC_50_) against VEGFR2, 4.0 nmol/L (27) versus 10 nmol/L and 90 nmol/L, respectively, while is inferior to the inhibition power of cabozantinib (IC_50_ 0.035) (28). Even if lenvatinib has been tested in ccRCC in the second line, in combination with everolimus, we have demonstrated its impressive activity as a single agent, in a highly pretreated patient. Patient selection could have contributed to the remarkable activity of lenvatinib, considering the response obtained with the previous lines of treatment. We relied on lenvatinib alone due to the risk of toxicity, considering the numerous previous lines of therapies, and we started at 20 mg as this is the standard dosage when lenvatinib is used in combination with pembrolizumab in renal cell cancer. AEs during lenvatinib were consistent with the drug profile and no complications (e.g., fistula) have been observed, especially in those tumor lesions included in the previous radiotherapy fields; rather, a significant and prolonged tumor shrinkage has been observed over time (Figure 1).

## Conclusions

We have proved the efficacy of multiple antiangiogenic agent sequences without cross-resistance, highlighting the pivotal role maintained by the angiogenic pathway even in the late phase of the disease. Following the dictum existing in oncology, “the best first”, one of the most potent antiangiogenic agents should be anticipated in the RCC patient journey.
